# Supplementary material for: Active intermixing of indirect and direct neurons builds the striatal mosaic
Source: Nat Commun. 2018 Nov 9;9:4725. doi: 10.1038/s41467-018-07171-4 (PMC6226429; doi:10.1038/s41467-018-07171-4)
Supplement: Supplementary file 1 — Supplementary Information [file 41467_2018_7171_MOESM1_ESM.pdf]

SUPPLEMENTARY FIGURES – Tinterri et al.

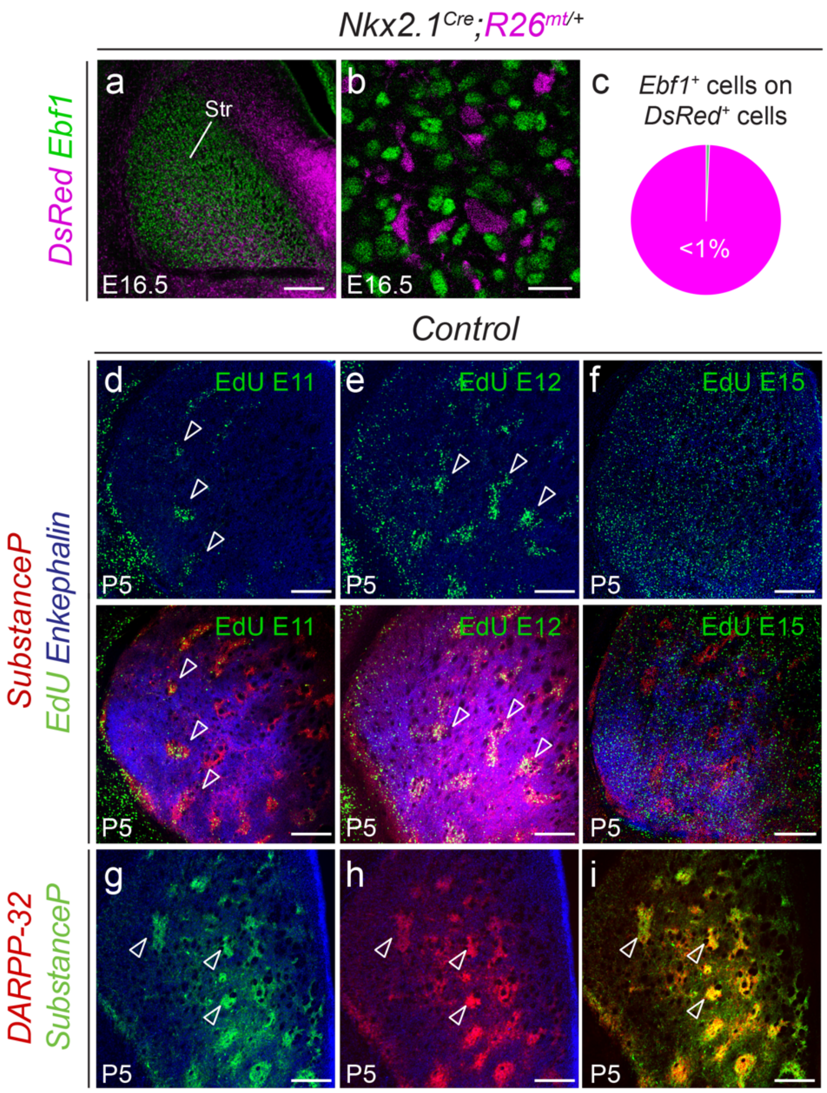

Figure S1. Developmental fingerprint of dSPN across striatal compartments

(**a-c**) Ebf1 protein is not detected in striatal neurons that are labelled by *Nkx2.1*^{Cre} recombination. (c) Quantification of DsRed+ striatal interneurons that are double-positive for Ebf1 (1,14±0,87%, n=3). (**d-i**) Birthdating with EdU unambiguously labels striosomes (arrowheads) at E11 (d) and E12 (e) and the matrix at E15 (f) thereby confirming the striosome/matrix specificity of Substance P and Enkephalin immunostaining at P5 (bottom panels) and DARPP-32 specificity in striosomes (g-i) (n=3 for each condition). Results presented as mean values ± standard deviation. Scale bar equals 250 μm. Str, striatum.

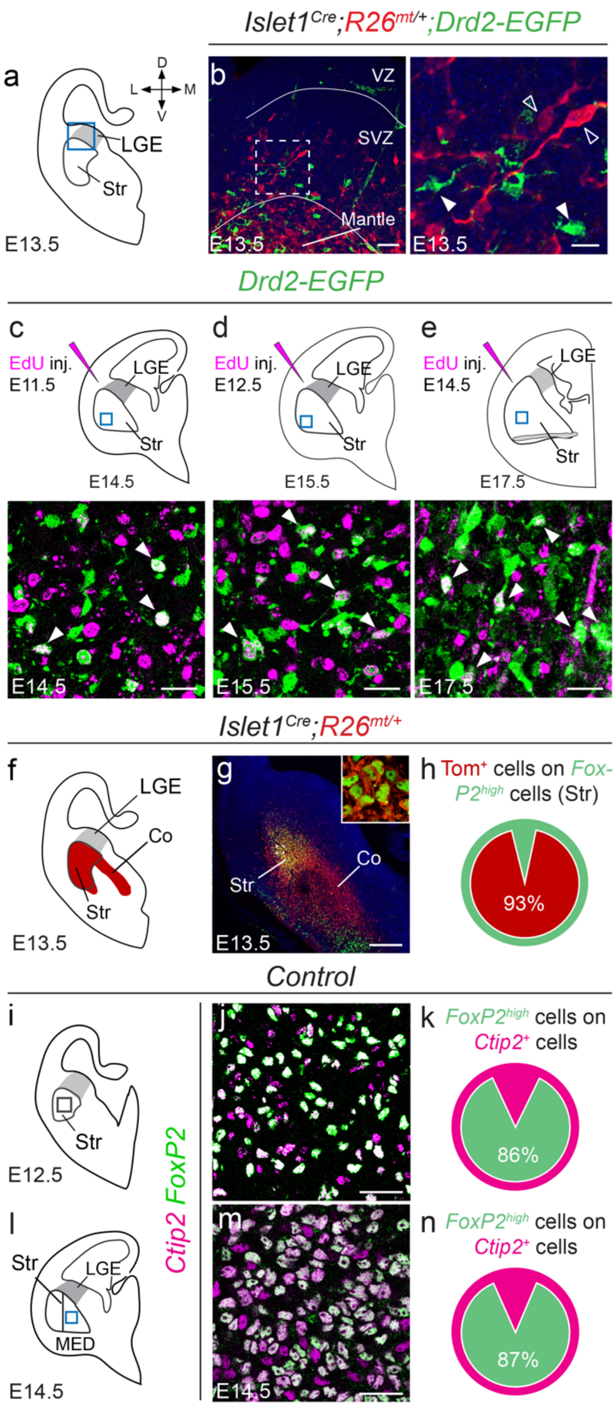

Figure S2. Drd2-EGFP expression and generation timecourse of early iSPN

(**a**) Schematic representation of E13.5 forebrain hemisection. The LGE subventricular zone (SVZ) shown in (**b**) is highlighted. (**b**) Low and high magnification of the LGE in E13.5 *Islet1*^{Cre}*;R26*$^{mt/+}$*;Drd2-EGFP* embryos shows that tdTomato+ dSPN (empty arrowheads) and GFP+ iSPN (full arrowheads) are already detected in the SVZ, before entering the striatal

mantle region. (**c**-**e**) iSPN are generated progressively. *Drd2-EGFP* embryos injected with EdU at E11.5 (c), E12.5 (d) and E14.5 and collected three days afterwards show co-labeling with EdU, indicating that iSPN are continuously generated. (**f**-**h**) The majority of striatal FoxP2^{high} cells are part of the Islet1 lineage (93±3% of all FoxP2^{high} cells) at E13.5. (**i**-**k**) The majority of Ctip2^{+} SPN in the striatal anlage at E12.5 express high level of FoxP2 (86±6% of all Ctip2^{+} neurons), confirming that at this stage dSPN are the most abundant population. (**l**-**n**) Similarly, in E14.5 medial striatum 87±4% of all Ctip2+ neurons are double-positive for FoxP2^{high}, confirming that at this stage the area contains mostly dSPN. At least n=3 mice for each stage. Results are presented as mean ± standard deviation. Scale bars equal 30 µm (b), 10 µm (high magnification of b), 25 µm (c-e), 200 µm (g) 50 µm (j,m), . Co, corridor; LGE, lateral ganglionic eminence; MED, medial striatum; Str, striatum; SVZ, subventricular zone; VZ, ventricular zone.

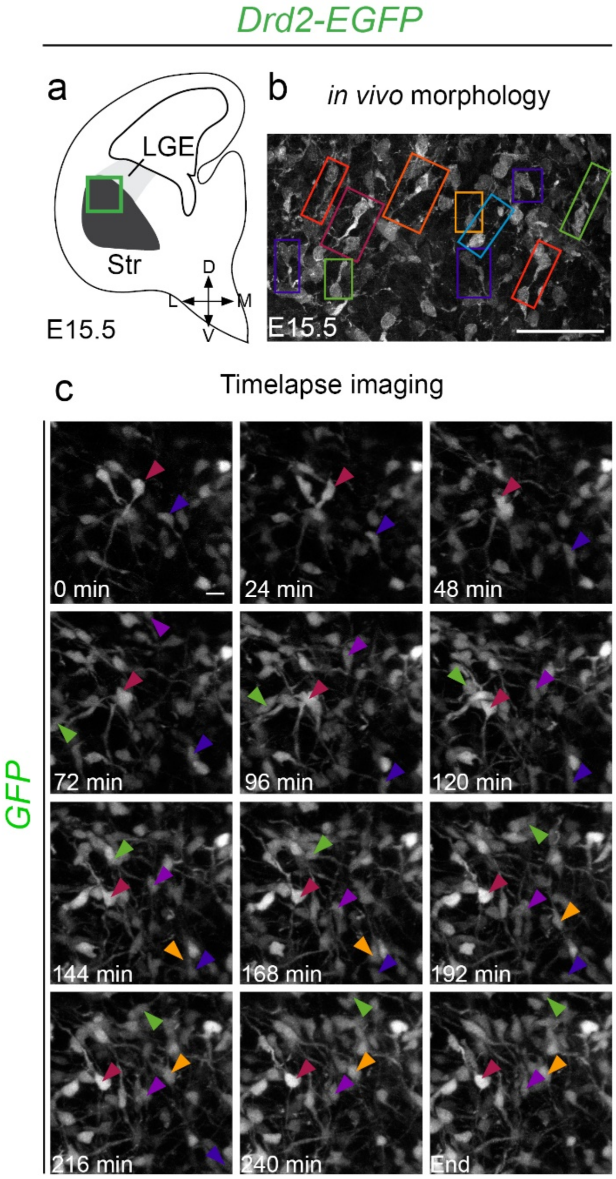

Figure S3. ***In vivo*** **and** ***ex vivo*** **multidirectional migration of iSPN**

(**a**) Schematic representation of E15.5 forebrain hemisection where the LGE and striatal primordium shown in (b) is highlighted. (**b**) E15.5 Drd2-EGFP+ iSPN morphology show different orientation, consistent with multidirectional migration *in vivo* (n=4). (**c**) Inset of timelapse shown in Fig. 3b; nearby iSPN migrate in different directions. Arrowheads track the movement of single cells. Scale bars equal 25 μm (b) and 10 μm (c). LGE, lateral ganglionic eminence; Str, striatum.

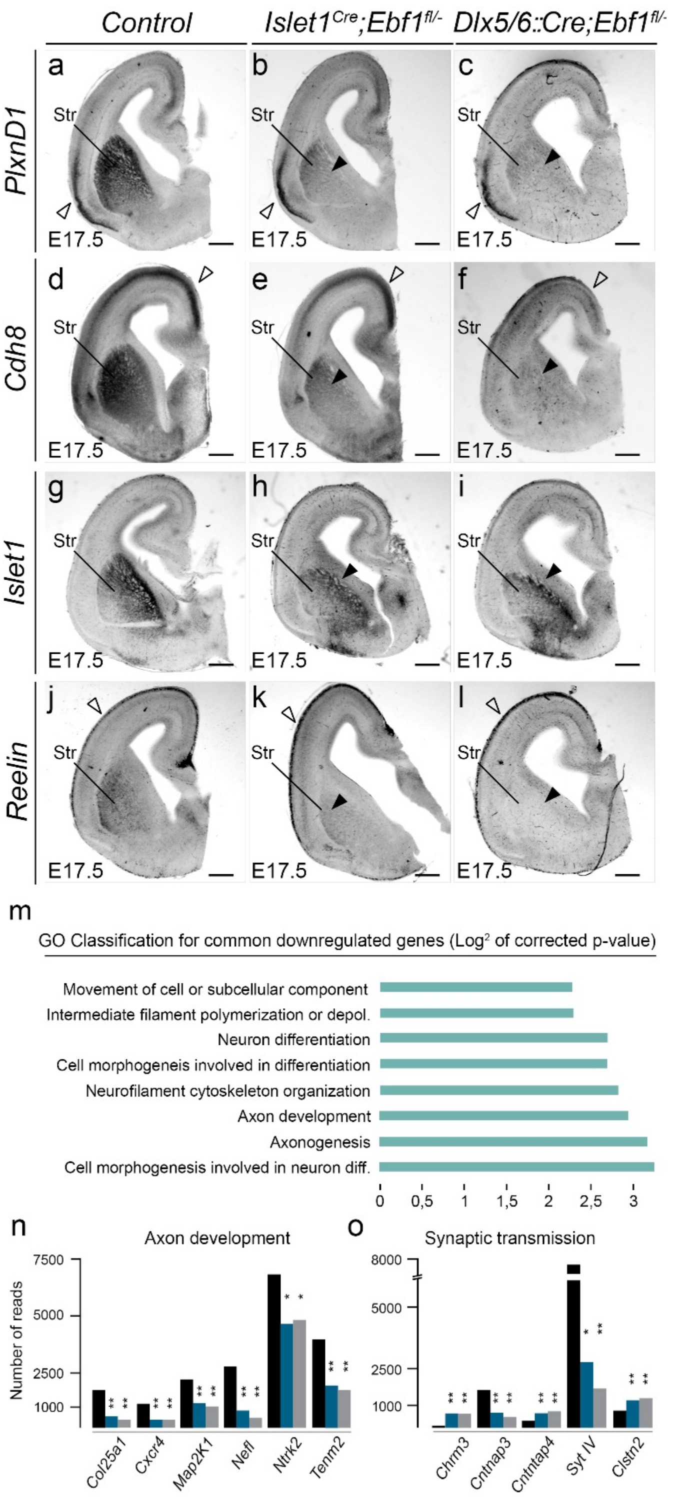

Figure S4. Modification of gene expression in *Ebf1* cKO

(**a-l**) *In situ* hybridization of selected genes from the RNAseq analysis shows a specific reduction in striatal expression (solid arrowhead) and preserved expression in other forebrain regions (open arrowheads) in E17.5 *Islet1^{Cre};Ebf1$^{fl/-}$* and *Dlx5/6::Cre;Ebf1$^{fl/-}$*

mutants (n=3 for each condition). (**m-o**) Gene Onthology (GO) analysis for genes downregulated in RNA-seq screening of *Islet1*^{Cre}*;Ebf1*$^{fl/-}$ and *Dlx5/6::Cre;Ebf1*$^{fl/-}$ embryos, compared to controls (>300 reads on average, adj. p<0.005), highlight dramatic alteration in genes associated with axon development (n) and synaptic transmission (o). Statistical comparison performed with DESeq 1.8.3 (see methods section); *indicates adjusted p-value <0.05: ** adjusted p-value<0.001. Scale bar equals 400 μm. Str, striatum.

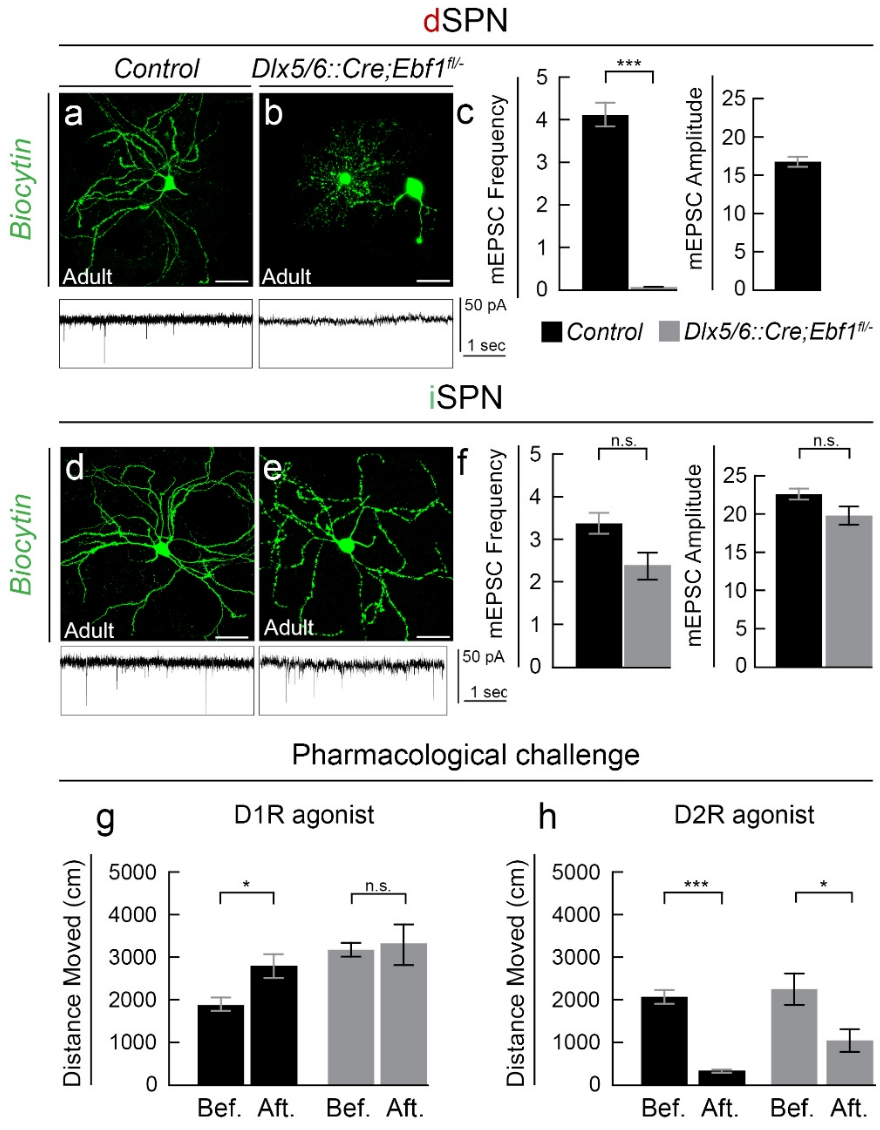

Figure S5. Indirect pathway activity is preserved in *Dlx5/6::Cre;Ebf1$^{fl/-}$* mice

(**a-c**) Patch-clamp recordings of dSPN in acute slices obtained from control (33 cells) and *Dlx5/6::Cre;Ebf1$^{fl/-}$;Drd2-EGFP* (14 cells) adult mice highlight a drastic decrease of mEPSC frequency in mutants (sample tracks in a-b, quantification of mEPSC in c). **(d-f)** Conversely, iSPN recorded in controls (27 cells) and *Dlx5/6::Cre;Ebf1$^{fl/-}$;Drd2-EGFP* (13 cells) slices show no relevant differences in mEPSC amplitude and frequency. mEPSC amplitudes could not be quantified in mutant dSPN because of the extremely low number of events recorded. Results are presented as mean values ± s.e.m. (**g-h**) Pharmacological challenge of adult *Dlx5/6::Cre;Ebf1$^{fl/-}$* mice show no response to D1R agonist SKF38393 (g) but normal

reaction to D2R agonist quinpirole (h), as shown by measurement of total distance travelled in 10 minutes in open field before (Bef.) and 40 minutes after (Aft.) injection of either drug ($n_{control}$=8 and n_{cKO}=8). Results are presented as mean values ± s.e.m. Two-tailed non-parametric Mann-Whitney U test was used for statistical comparison. * indicates p-value <0.05, ***p-value <0.0001.

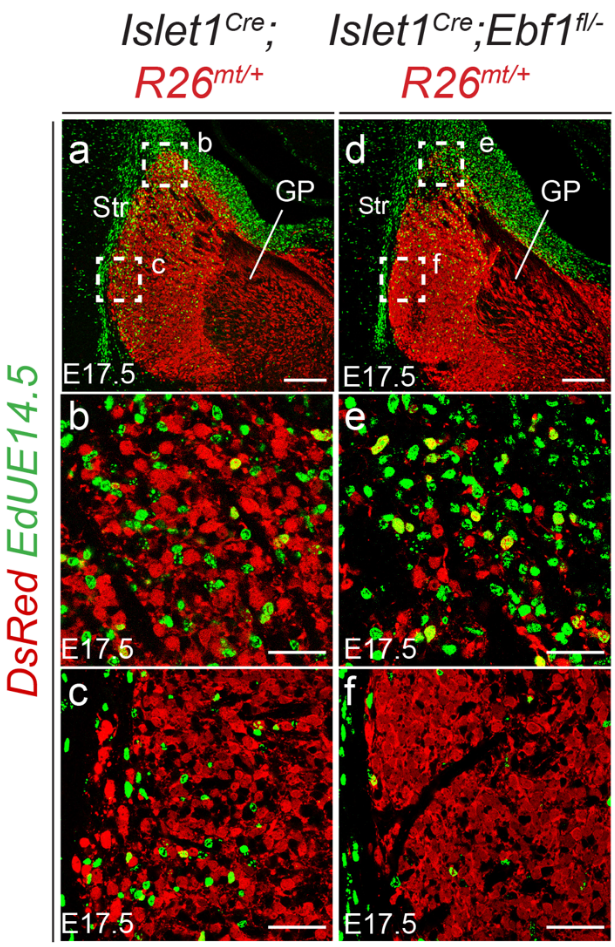

Figure S6. Progression of late-born iSPN in the striatum is impaired in *Ebf1* cKO

(**a-f**) Coronal striatal hemisections in E17.5 *Islet1^{Cre};Ebf1$^{fl/-}$;R26$^{mt/+}$* and control *Islet1^{Cre};R26$^{mt/+}$* littermates injected with 5-ethynyl-2'-deoxyuridine (EdU) at E14.5. Distribution of matrix iSPN (*EdUE14*$^{+}$ and tdTomato^{-}) is altered in cKO, with more cells located dorsally (b,e) and less ventrally (c-f) indicating a delay in iSPN migration (n=3 for each condition). Scale bars equal 200 µm (a,d), 50 µm (b,c,e,f). GP, globus pallidus; Str, striatum.

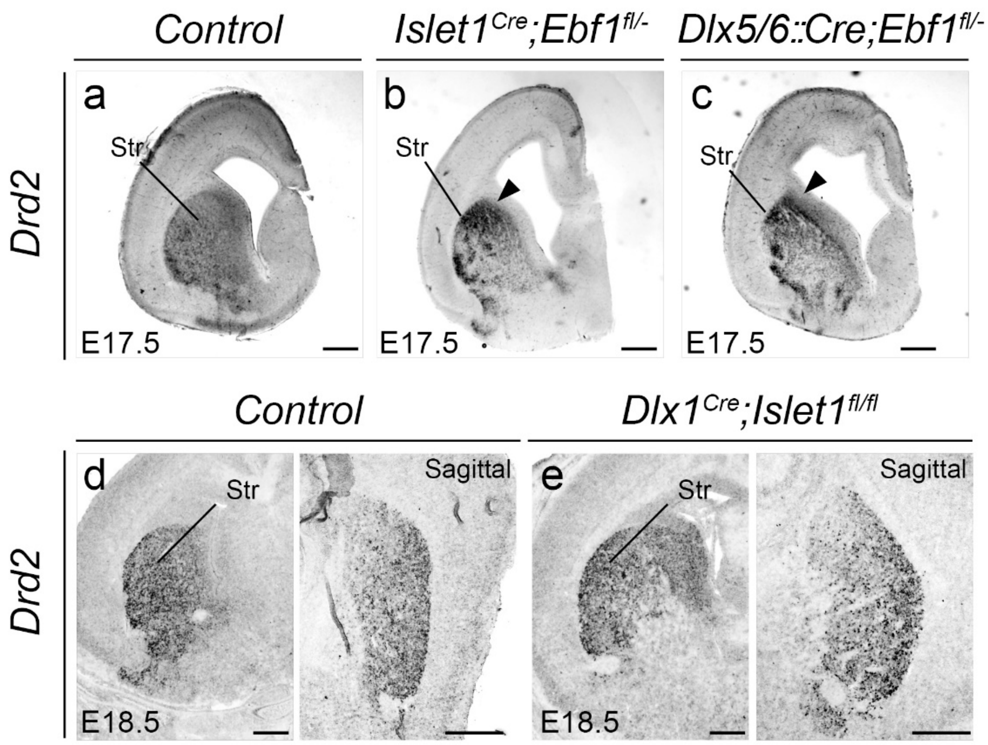

Figure S7. iSPN distribution phenotype is specific to *Ebf1* cKO

(a-c) Coronal striatal hemisections of E17.5 control, *Islet1*^{Cre};*Ebf1*$^{fl/-}$ and *Dlx5/6::Cre;Ebf1*$^{fl/-}$ brains. The distribution of Drd2 is dramatically altered in cKO with a clear accumulation in the dorsal part of the striatum (arrowhead) (n=3 for each condition). **(d-e)** Coronal and sagittal striatal hemisections of E18.5 control and *Dlx1*^{Cre};*Islet1*$^{fl/fl}$ brains. The distribution of Drd2 is not dramatically altered indicating that dSPN control of iSPN migration and intermix depends specifically on Ebf1 expression (n=3 for each genotype). Scale bar equals 400 µm. Str, striatum.

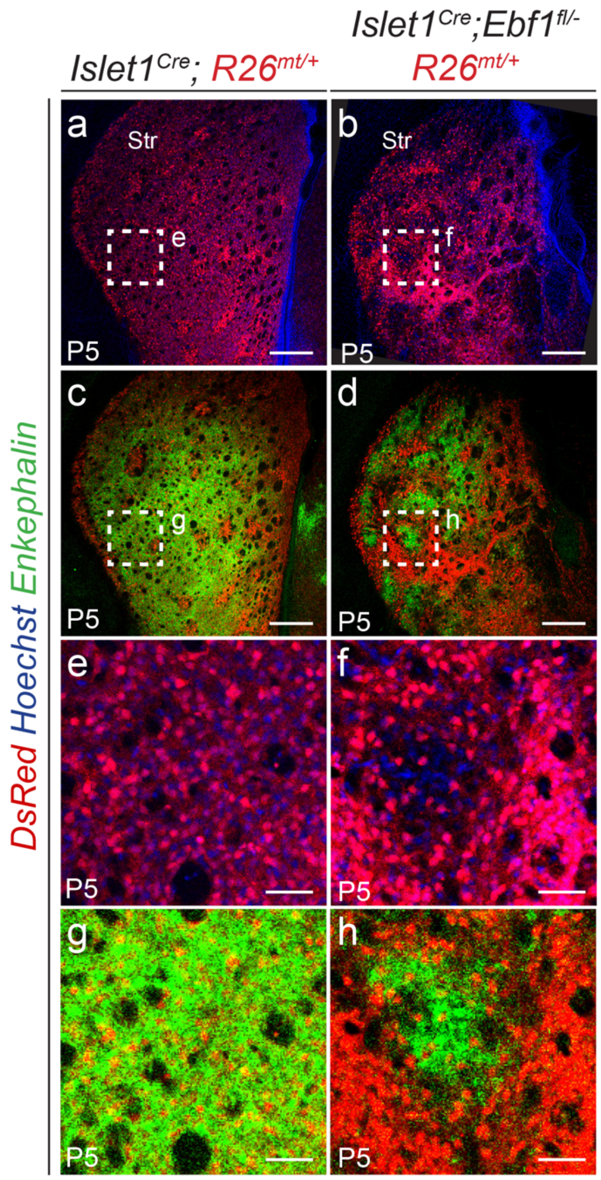

Figure S8. Defective distribution of dSPN in the postnatal matrix of *Ebf1* cKO

(a-h) Coronal striatal hemisections in P5 *Islet1*^{Cre}*;Ebf1*$^{fl/-}$*;R26*$^{mt/+}$ and control *Islet1*^{Cre}*;R26*$^{mt/+}$ mice. dtTomato+ dSPN distribution is altered in the cKO (a-b and c-d) as these cells form aggregates in Enkephalin-poor areas (f,h) instead of being homogeneously distributed in the Enkephalin^{high} matrix (e,g) (n=3 for each condition). Scale bar equals 200 µm (a-d), 50 µm (e-h).

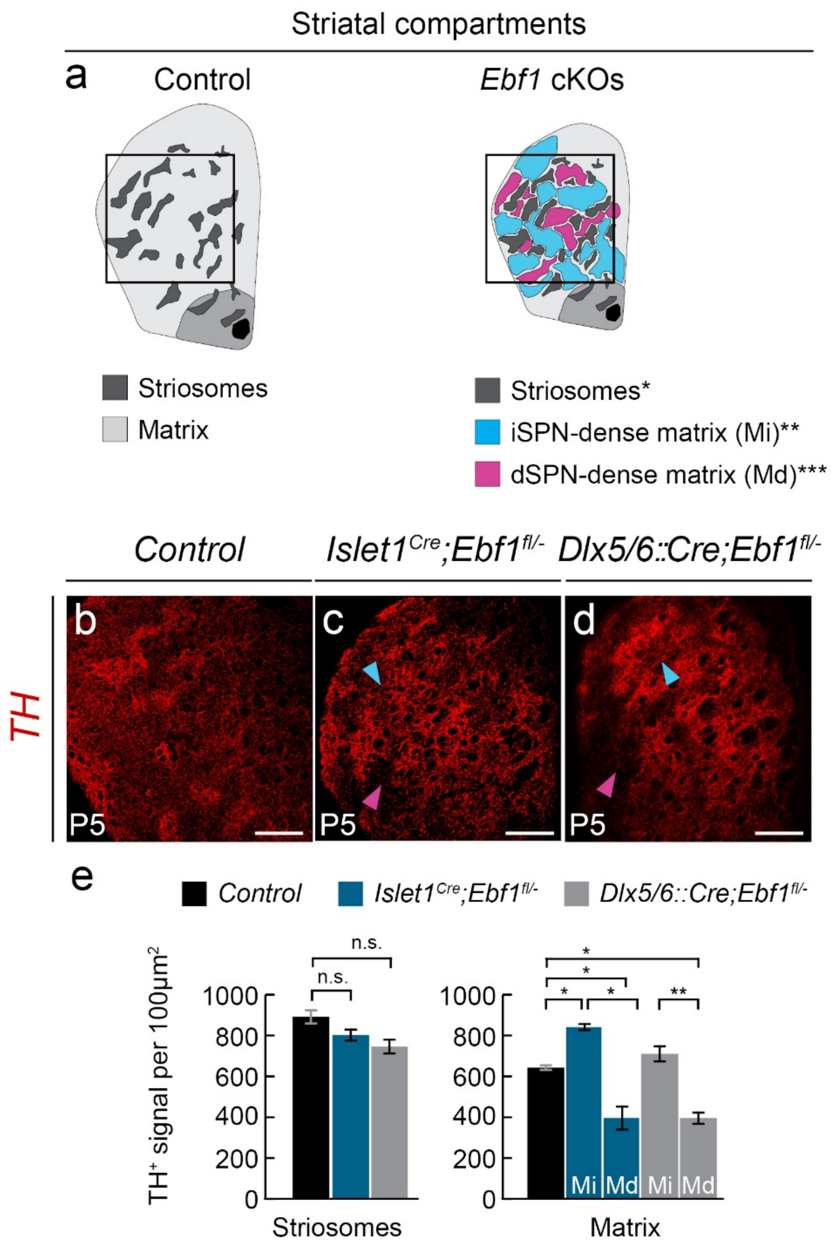

Figure S9. ***Ebf1*** **deletion affect dopaminergic input to the striatum**

Schematic representation of striatal compartments at P5 in controls and *Ebf1*. In controls, striosomes are defined by high levels of DARPP32 expression (DARPP32^{high}) and the matrix by high levels of Enkephalin expression (Enk^{high}). In *Ebf1* cKOs the matrix is parcelled into DARPP32^{low};Enk^{high} areas, enriched in iSPN (iSPN-dense matrix or Mi, cyan arrowheads in (c) and (d)) and DARPP32^{low};Enk^{low} areas populated by dSPN (dSPN-dense matrix or Md, magenta arrowheads in (c) and (d)). (**b-e**) TH+ dopaminergic inputs were not significantly

different in striosomes between controls (b) and either *Islet1*$^{Cre/+}$*;Ebf1*$^{fl/-}$ (c, p=0.17) and *Dlx5/6::Cre;Ebf1*$^{fl/-}$ (d, p=0.11) mice, as quantified in (e). Conversely, iSPN-rich (i) matrix receives significantly more input compared to control matrix (p=0.02 for *Islet1*$^{Cre/+}$*;Ebf1*$^{fl/-}$and p=0.04 for *Dlx5/6::Cre;Ebf1*$^{f/-}$), while dSPN-rich (d) matrix receives significantly less dopaminergic innervation compared to both control matrix (p=0.01 for *Islet1*$^{Cre/+}$*;Ebf1*$^{fl/-}$and p=0.02 for *Dlx5/6::Cre;Ebf1*$^{f/-}$) and to iSPN-rich regions (p=0.01 for *Islet1*$^{Cre/+}$*;Ebf1*$^{fl/-}$and p=0.003 for *Dlx5/6::Cre;Ebf1*$^{f/-}$). This indicates that matrix dSPN are not properly innervated by dopaminergic input in *Ebf1*cKOs. Results are presented as mean values ± s.e.m. Two-tailed non-parametric Mann-Whitney U test was used for statistical comparison. * indicates p-value <0.05, ** p-value <0.01. Scale bar equals 250 µm.

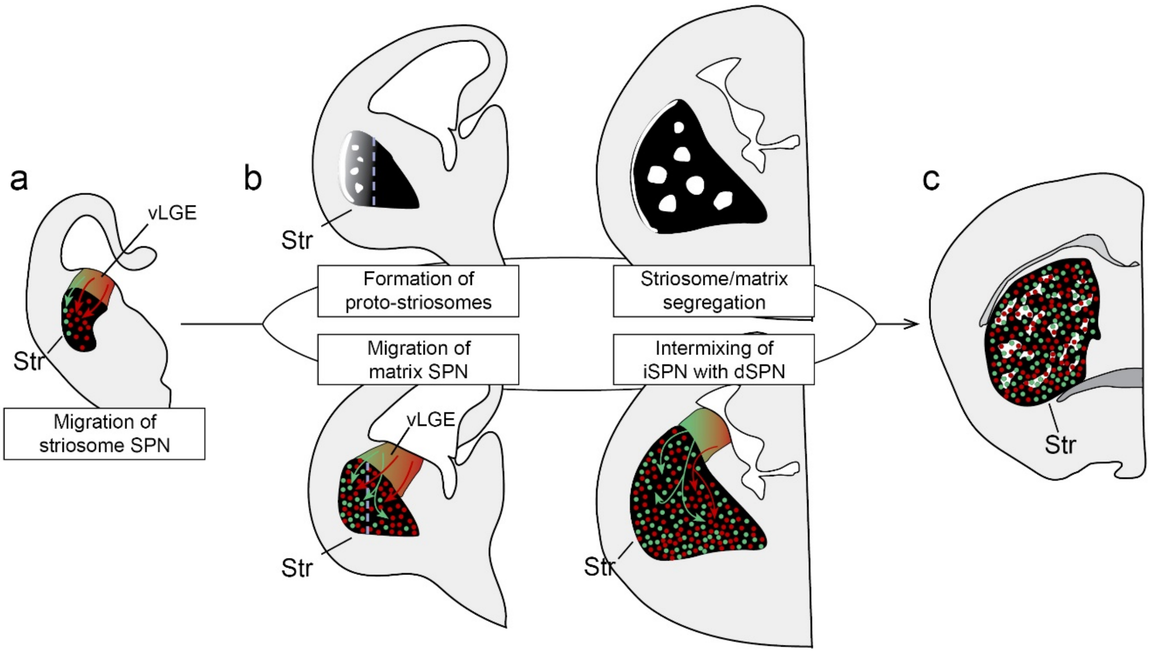

Figure S10. Working model for the development of striatal mosaic

(**a**) During the earliest stages of striatal development (up to around E13), fate-committed dSPN (mostly) and iSPN that will form striosomes migrate in the striatal anlage. (**b**) At mid striatogenesis, presumptive striosomes containing early-born SPN start forming in the lateralmost part of the striatum, while the structure progressively expands by virtue of the migration of later-born dSPN and iSPN that will form the striatal matrix. In the medial part of the striatum, initially containing mostly dSPN, iSPN migrating from the LGE SVZ intermix with dSPN. In parallel, early-born and late-born SPN segregate from each other to form striosome and matrix compartments. (**c**) After birth, intrastriatal migration stops, followed by the refinement of intra and extra striatal connectivity. Str, Striatum; SVZ, subventricular zone; vLGE, ventral part of the lateral ganglionic eminence.
