# Supplementary material for: Active intermixing of indirect and direct neurons builds the striatal mosaic
Source: Nat Commun. 2018 Nov 9;9:4725. doi: 10.1038/s41467-018-07171-4 (PMC6226429; doi:10.1038/s41467-018-07171-4)
Supplement: Supplementary file 8 — Description of Additional Supplementary Files [file 41467_2018_7171_MOESM8_ESM.docx]

**Title:** Supplementary Movie 1
**Description:** This movie is a 240-minutes video recording of a E15.5 Drd2-EGFP+ coronal acute slice showing multidirectional migration of GFP+ iSPN within the striatum and is representative of the different videos used to analyze iSPN migration in Figure 3. Each frame in the video is obtained by stacking 100 z-frames taken at a 1μm interval; stacks were imaged every 6 minutes.

**Title:** Supplementary Movie 2.
**Description:** This movie is another example of 240-minutes video recording of a E15.5 Drd2- EGFP+ coronal acute slice showing multidirectional migration of GFP+ iSPN within the striatum, and is representative of the different videos used to measure iSPN migration in Figure 7. Each frame in the video is obtained by stacking 100 z-frames taken at a 1μm interval; stacks were imaged every 6 minutes.

**Title:** Supplementary Movie 3.
**Description:** This movie is a 240-minutes video recording of a E15.5 Islet1Cre/+; Ebf1fl/-; Drd2- EGFP+ coronal acute slice showing multidirectional migration of GFP+ iSPN with reduced speed compared to controls, and is representative of the different videos used to measure iSPN migration in Figure 7. Each frame in the video is obtained by stacking 100 z-frames taken at a 1μm interval; stacks were imaged every 6 minutes.

**Title:** Supplementary Movie 4.
**Description:** This movie is a 240-minutes video recording of a E15.5 Dlx5/6::Cre; Ebf1fl/-; Drd2- EGFP+ coronal acute slice showing multidirectional migration of GFP+ iSPN with reduced speed compared to controls, and is representative of the different videos used to measure iSPN migration in Figure 7. Each frame in the video is obtained by stacking 100 z-frames taken at a 1μm interval; stacks were imaged every 6 minutes.

**Title:**  Supplementary Data 1
**Description:** Table contains the result of differential analysis for gene expression in control versus Islet1Cre;Ebf1fl/- conditions. The analysis of raw FASTQ files (obtained from Illumina sequencer) was performed using the Eulsan pipeline. The columns of the expression file are described below.

**Title:** Supplementary Data 2
**Description:** This table contains the result of differential analysis for gene expression in control versus Dlx5/6::Cre;Ebf1fl/- conditions. The analysis of raw FASTQ files (obtained from Illumina sequencer) was performed using the Eulsan pipeline.
